# Reversing the eyes and reverse perspectives: Pseudoscopic amplification of reverspectives

**DOI:** 10.1177/20416695231215406

**Published:** 2023-11-16

**Authors:** Nicholas J. Wade, Patrick Hughes

**Affiliations:** Department of Psychology, University of Dundee, Dundee, UK; 72 Great Eastern Street, London EC2A 3JL, London, UK

**Keywords:** reverse perspective, stereoscopic and pseudoscopic photographs, anaglyphs

## Abstract

Stereoscopic photographs of works in reverse perspective do not reveal their three-dimensional structure whereas pseudoscopic photographs enhance the apparent depth effects.

The reverse perspectives of Patrick Hughes are in relief but are painted to appear like conventional flat pictures; those parts that protrude from the picture plane are pictorially distant, or in reverse perspective (Papathomas & Hughes, 2019; Rogers & Hughes, 2023; Wade & Hughes, 1999). They have been given the name “Reverspectives” and their relevance to visual science has been appreciated. It is of interest that the three visual scientists above who have published with Patrick Hughes have adopted different interpretations of Reverspectives! Many of the perceptual characteristics of Reverspectives have been determined by Thomas Papathomas and Brian Rogers (see Papathomas, 2002, 2017; Papathomas & Hughes, 2019; Rogers, 2017; Rogers & Gyani, 2010; Rogers & Hughes, 2023).

Most artistic styles that set out to fool the eye (*trompe l’oeil*) attempt to make a flat pictorial surface look three-dimensional (3-D) whereas Reverspectives make the 3-D structure appear flat. Movements of the observer result in relative motions of the pictorial image that are not seen with flat pictures. These distortions occur with binocular observation and over a wide range of viewing distances. That is, binocular disparities signifying the 3-D structure of the works do not override the perspectival illusion until the observer is close to them. The distance of the observer from the works that results in the change in perception from flat and fluid to solid and stable is greater with binocular than monocular viewing (Papathomas, 2002; Rogers & Hughes, 2023). It is not possible to obtain these experiences with photographs of the works, an example of which is shown in Figure 1, but some relative motions can be displayed with videos of rotations around Reverspectives (see https://www.patrickhughes.co.uk/video/).

**Figure 1.**
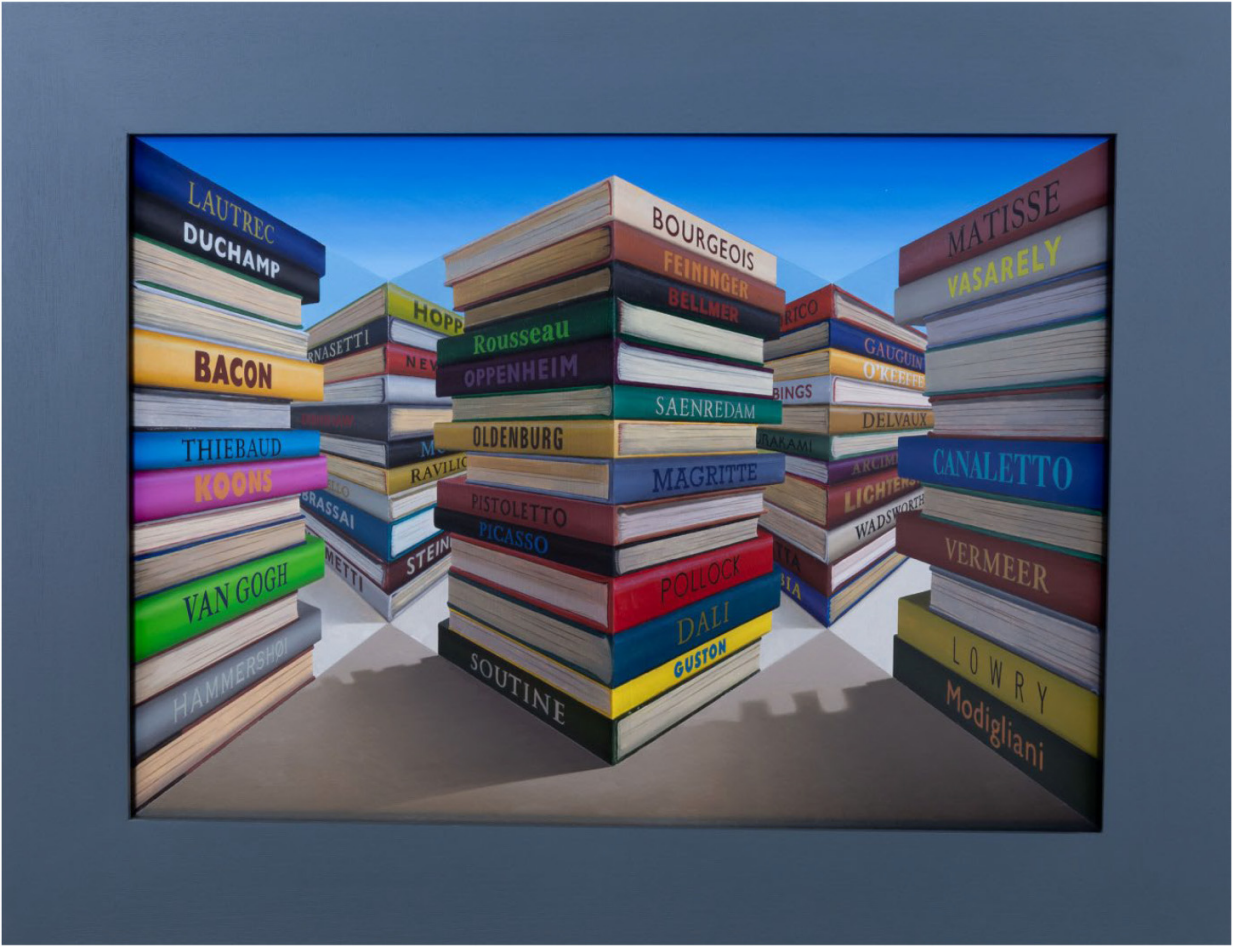
A photograph of *Catalogues* (2021) by Patrick Hughes.

Although the distortions of visual space induced by the works do survive binocular viewing up to short separations between the eyes and the protrusions, it might be feasible to expose the 3-D aspects of the works with photographs taken from laterally separated positions. Stereoscopic photographs are usually taken with twin lensed cameras or paired cameras mounted on specially designed rigs but they can be derived from spatially separated views from a single camera taken sequentially. No matter how they are taken they require a stereoscope of some kind to view them. The stereoscopic photographs shown here are anaglyphs which are viewed through red/cyan filters. The conventional arrangement is for the red filter to be in front of the left eye (LE) and the cyan filter for the right eye (RE). An example of an anaglyphic stereoscopic combination of a 3-D scene is shown in Figure 2. The apparent depth is compelling with the conventional combination of red/LE and cyan/RE. However, with a reversal of the filters (cyan/LE and red/RE) the apparent depth is diminished but not reversed. Many examples of the comparative effects of stereoscopic and pseudoscopic viewing of anaglyphs can be seen in Wade (2023).

**Figure 2.**
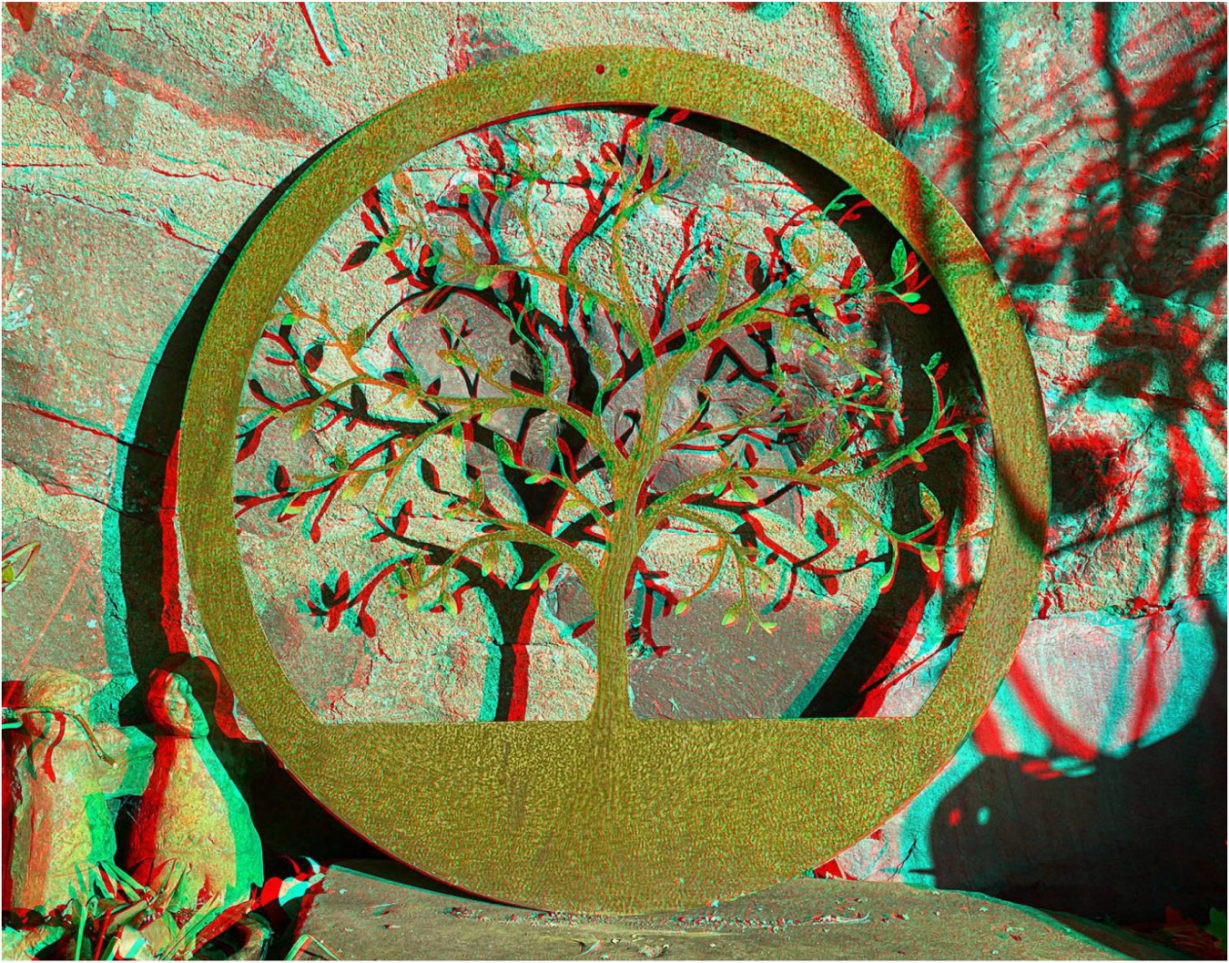
*Tree shadows* by Nicholas Wade.

Wheatstone (1838) referred to reversing the sign of disparities as “conversions of relief” and he later devised a pseudoscope for producing the effect. Wheatstone (1852) described the difficulties associated with seeing the reversed disparities when he directed the pseudoscope to actual objects. This applies to photographs, too (Brewster, 1856; Jastrow, 1900; Wallin, 1905).

Figure 3 is an anaglyph of *Catalogues*. Viewing it in the conventional way (red/LE, cyan/RE) yields disparities that should reveal the physical structure of the work but it does not. Pictorial perspective is more powerful than disparity, as has been shown previously. Unlike conventional stereo photographs of objects, reversing the filters (cyan/LE and red/RE) enhances rather than diminishes the apparent depth. That is, a stereoscopic photograph competes with the perspectival depth whereas a pseudoscopic photograph cooperates with it: if the disparities correspond to the perspective apparent depth will be increased. Put another way, it is the painted perspective, made in reverse, whose apparent depth is increased. It is difficult to think of any structure other than Reverspectives for which this would apply.

**Figure 3.**
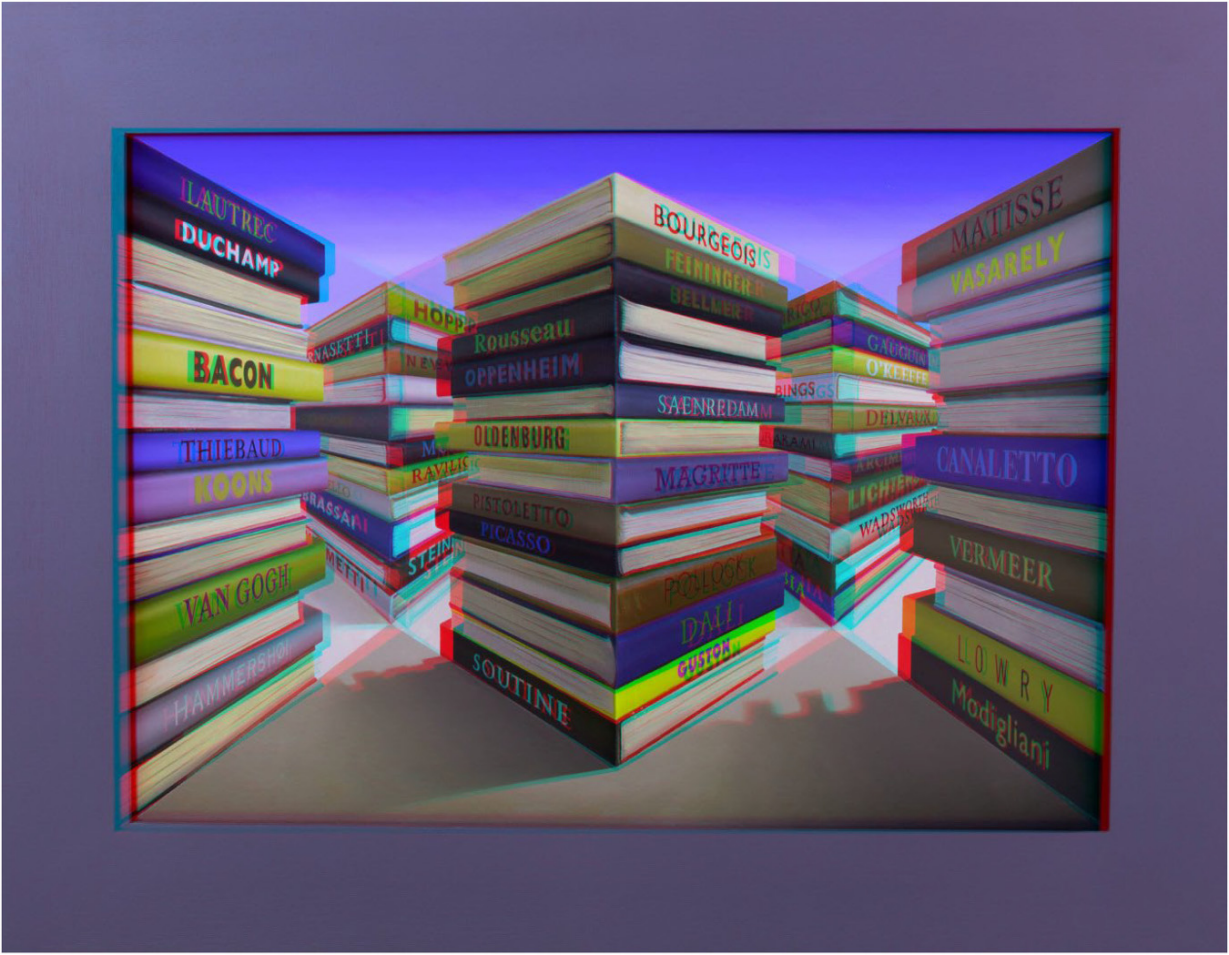
An anaglyph of *Catalogues*.

It remains the case that there is no adequate substitute for seeing the Reverspectives in 3-D. Only then, with head movements, are the fluid relative motions within the pictorial space adequately experienced.
